# Does early drug use‐related police contact predict premature mortality and morbidity: A population register‐based study

**DOI:** 10.1111/dar.13416

**Published:** 2021-12-15

**Authors:** Noora Ellonen, Joonas Pitkänen, Bryan L. Miller, Hanna Remes, Mikko Aaltonen, Atte Oksanen, Pekka Martikainen

**Affiliations:** ^1^ Faculty of Social Science Tampere University Tampere Finland; ^2^ Population Research Unit University of Helsinki Helsinki Finland; ^3^ Department of Sociology, Anthropology and Crimianl Justice Clemson University Clemson USA; ^4^ Law School University of Eastern Finland Joensuu Finland

**Keywords:** drug use, adolescence, mortality, patient care, morbidity

## Abstract

**Introduction:**

The aim was to analyse whether age at first drug offense predicts premature mortality and morbidity due to substance use and violence among adolescents and young adults.

**Methods:**

A prospective longitudinal register‐linkage study based on a total population sample from Finland including individuals born between 1987 and 1992 and aged 15–25 years during follow‐up in 2002–2017 (*n* = 386 435). Age‐specific rates of deaths and health‐care admissions (morbidity) during a 5‐year follow‐up were calculated from the first drug offense. Cox regression models were used to estimate differences in mortality and morbidity at ages 21–25.

**Results:**

Of all 15‐ to 20‐year‐olds, 1.4% (*n =* 5540) have had a police contact. The 5‐year mortality rates (per 1000 person‐years) among those with first drug offense at ages 15–16 was 2.92 [95% confidence interval (CI) 1.56–6.18], and 5.26 (CI 4.00–7.07) and 5.05 (CI 4.06–6.38) at ages 17–18, and 19–20, respectively. The rates of morbidity varied between 61.20 (CI 52.43–71.76) and 87.51 (CI 82.11–93.33). Both mortality and morbidity rates were over 10 times higher than among the general population. In models adjusted for family background, first police contact at an early age (15–16) did not increase the risk of mortality at ages 21–25 compared with first police contact at ages 17–18 (hazard ratio 1.55, CI 0.77–3.09) or 19–20 (hazard ratio 1.52, CI 0.78–2.98). The results were similar for morbidity.

**Discussion and Conclusions:**

Adolescents with drug‐related police contacts have high risk of mortality and morbidity due to substance use and violence regardless of age of first contact.

## Introduction

Individuals who use drugs have an increased risk of both overall premature mortality and mortality directly related to acute and chronic drug use [1, 2, 3, 4]. Increased risk of premature mortality is moderated by a number of factors, including the types of substances used, mental health and risky behaviour [3, 4, 5]. In addition, adolescent onset drug use has been shown to be associated with increased risk of premature mortality [6]. However, evidence of this relationship is limited and the only empirical research supporting this assumption is a study by Clark and colleagues [6], where they analysed the association between early onset drug use before age 18 and premature mortality based on a longitudinal sample of 870 adolescents with substance use disorders from clinical programs and community sources finding 21 deaths before the age of 25. Thus, it remains unclear whether the association between the age of onset and higher risk of premature mortality is linear (younger onset—higher risk). Regarding psychiatric morbidity, early drug use onset has been shown to increase the risk of drug dependence [7], the risk of schizophrenia [8], as well as psychosocial problems [9], more than later drug use onset.

Most of these studies used the age of 18 as the cutoff for early versus late onset and are unable to answer the question if the risks of mortality and morbidity increase when the age of drug onset decreases among those under 18. In addition, the methodological designs in these studies on morbidity limit their generalisability, since they are based on clinical samples of individuals in treatment [9] or cross‐sectional survey data with retrospective self‐report measures [7, 8], of which the latter may suffer from non‐response bias and might also fail to reach marginalised populations, such as drug users. Given the many challenges in collecting generalisable data on adolescents' drug use, these methodological limitations are understandable. An alternative methodology, although not without its own limitations, is to use population level studies and administrative register data.

Administrative data might cover hard‐to‐reach populations, such as heavy drug users, more comprehensively than surveys, especially in the Finnish context where each individual permanently residing in Finland is issued a personal identification number and included in this data. Also, administrative register data enables prospective designs without recall bias [10] and can bring new insights to the study of health consequences of early drug use onset, as it has done for the effects of early onset alcohol use. A Swedish longitudinal register‐based study [11] found there is no clear connection between the earlier onset of alcohol use and premature risk of death when compared to later onset, although studies based on cross‐sectional designs suggested such effects [12]. Further, Kendler and his colleagues [13] analysed the association between drug use and mortality based on nationwide register data showing a strong association between registry‐ascertained drug use and premature mortality from both non‐medical and medical causes. Excess mortality was explained with both indirect effects—characteristics of drug‐abusing persons—and direct effects from the drug use itself. Official register data have not been used to analyse the effect of early onset drug use on mortality or morbidity.

This study investigates the association between early drug use‐related police contact and the risk for mortality and morbidity due to substance use and violence by employing Finnish administrative register data including all deaths (mortality) and health‐care admissions (morbidity) of mental and behavioural disorders due to psychoactive substance use, poisoning by drugs, medications, alcohol, intentional self‐harm and assaults based on the International Classification of Diseases (ICD‐10). Early drug use‐related police contact refers to the relative early age in which an adolescent first comes into contact with authorities. Because register data only includes administrative information reported to authorities, it does not include accurate measures of actual drug use onset. First drug use‐related police contact is thus utilised as a proxy measure of drug use onset. Although this strategy is not without limitations, in Nordic countries police registers describe well overall drug use [4] and employing this measure alleviates concerns over recall bias. Further, by investigating the linear relationship between early drug use onset and health consequences, targeted interventions can be better aligned towards adolescent groups most as risk.

First, the study describes the background of adolescents with early and later onset in drug‐related police contacts. Second, the study investigates whether early onset in drug‐related police contacts predicts premature mortality or morbidity due to substance use and violence.

## Methods

### Participants

The study was based on a total population sample of all children born in Finland between 1986 and 2000. For all children and their parents, annual population census data were linked with suspected criminal offenses, deaths, and inpatient and specialised outpatient care episodes using personal identification numbers. We limited our analyses to those born between 1987 and 1992 and residing in Finland at the start of the year they turned 15 (*n* = 387 747). We excluded children with missing data on mothers (*n* = 1037) or parental education (*n* = 275). We followed the remaining 386 435 individuals for suspected criminal offenses of drug use from age 15 to 20. We focused on those adolescents who had at least one police record of drug use during this time (*n* = 5540) and followed them for mortality and health‐care admissions for a 5‐year period from the first offense onwards and between ages 21–25.

### Measures

#### Age at first drug offense

Data on drug use‐related police contacts came from police registers and refers to the first suspected criminal offense for unlawful use of narcotics (criminalised in Finnish Criminal Code 50:2a§) between ages 15 and 20. In Finland, several types of drug use is criminalised outside of medical prescription including cannabis, stimulants and opioids. The most common drug offense is for the unlawful use of narcotics. This offense indicates that an individual was caught with a small amount of a drug considered for personal use, not for selling purposes. The most common drugs among adolescents and young adults in Finland are cannabis and amphetamine [14, 15]. The age for criminal responsibility in Finland is 15 and offenses committed under 15 years of age do not result in a criminal process but result in social and health interventions [16]. A criminal offense in later adolescence (<18 years) also results in automatic interventions from social services in addition to a criminal process. The age at first drug use‐related offense was grouped into 15–16, 17–18 and 19–20 years in the analyses.

#### Premature mortality and morbidity due to substance use and violence

We included all deaths (mortality) and health‐care admissions (morbidity) of mental and behavioural disorders due to psychoactive substance use, poisoning by drugs, medications, alcohol, intentional self‐harm and assaults based on the ICD‐10 (ICD‐codes in Appendix A). Data on mortality came from the national cause‐of‐death register where the determination of the cause of death is based on the medical or forensic evidence and issued by a physician. Data on morbidity came from the Finnish Care Register for Health Care that covers all institutions providing hospital‐level care. The register includes inpatient admissions (overnight stays) as well as outpatient care by medical specialists. The register does not cover visits to primary health care. We considered only the first admissions to care during the follow‐up period.

#### Background factors

To account for known socioeconomic differences in mortality and morbidity due to substance use and violence, as well as drug use related police contacts, we controlled for parental education. The variable was based on the highest completed degree by either biological parent and measured at child's age 12–14 and classified into tertiary (13 years or more), secondary (11–12 years) and basic education (<10 years). Family structure at age 14 was also included as a control. The variable was based on the child's living arrangements at age 14 and classified into: (i) two‐parent family; (ii) single‐parent family; and (iii) outside families. Both, parental education and family structure have shown to be associated with adolescent drug use [17]. Finally, in order to capture possible differences by gender, cohort and location, the analyses also included controls for sex, birth year and university hospital catchment area (*n* = 5).

### Statistical methods

#### Rates of death and health‐care admissions

We conducted a 5‐year follow‐up for deaths and care episodes due to substance use or violence for adolescents who had at least one police record of drug use from age 15 to 20 from the start of the year of their first police record. Adolescents were censored at emigration or death for other causes than substance use or violence. We calculated rates of mortality and morbidity per 1000 person‐years by the age at first suspected criminal offense for drug use, and used Kaplan–Meier survival curves to illustrate the probability of survival during the follow‐up. As there were multiple annual records per individual, we used cluster‐robust standard errors while calculating the rates. For comparative purposes, and to assess whether any excess risks by age at first offense showed a difference from the general age‐patterning of mortality, we also calculated age‐specific rates of health‐care admissions and deaths for the general population born in 1987–1992 for the 5‐year period 2007–2011, using age in 2007 (15–16/17–18/19–20) as our grouping variable.

#### Cox proportional hazards models

To further elaborate the age‐related patterns, we estimated a series of Cox proportional hazards regressions. Instead of starting the follow‐up period from the year of the first police contact, we conducted a 5‐year follow‐up for both deaths and health‐care admissions from age 21 to age 25, a design that allowed us to include the effect of age in the baseline hazard by using age as the follow‐up time. This mortality analysis is subject to a modest degree of ‘survivor’ bias, since the design includes only those alive at the start of the year they turn 21 (see details in results section). The subjects were censored at outcome of interest (death or health‐care admission), emigration, or at the end of the year of turning age 25.

We first present crude hazard ratios for each age group (model 0), then a model adjusted for birth year, sex and university hospital specific catchment area (model 1), and finally a model further adjusted for parental education and family structure (model 2). We use the same grouping of age at first drug use‐related police contact as above. We run the models including those 21–25‐year olds in the general population without police drug‐related record between age 15 and 20 for comparative purposes. We repeated the above procedure using health‐care admissions as the outcome event. In all the Cox models, we used cluster‐robust standard errors to account for possible correlation between siblings in the data. Mother's identification number was used to identify siblings.

## Results

This work employs the innovative use of longitudinal administrative register data and was not pre‐registered, therefore the results are exploratory. Of all 15‐ to 20‐year‐olds, 1.4% (5540) had a drug‐related police contact. The majority of these adolescents were males. The proportion of females was only 28% among the youngest drug user group (ages 15–16) and between 20% and 24% in other age groups. Among all age groups, the share of those whose parents had only basic education (15–19%) was significantly higher compared with the total population (7%) and a higher proportion did not live in families (6–9%) compared with the general population (1.5%). Over a third of 15‐ to 16‐year‐olds and more than 40% of older age groups had more than one police contact due to a drug offense during the 5‐year follow‐up since the first offense (Table 1).

**Table 1:** Distributions of baseline variables and the number of follow‐up years with at least one drug use‐related offense by age at first drug use‐related police contact and for the general population

	15–16 at first drug use offense		17–18 at first drug use offense		19–20 at first drug use offense		General population aged 15–20 in 2007	
	*n*	%	*n*	%	*n*	%	*n*	%
*Parental education*								
Basic	118	19.03	324	17.44	451	14.73	28 390	7.37
Secondary	289	46.61	892	48.01	1481	48.37	162 648	42.24
Tertiary	213	34.35	642	34.55	1130	36.90	193 982	50.38
*Family structure at age of 14*								
Not in family	56	9.03	119	6.40	183	5.98	5860	1.52
Two‐parent	320	51.61	1043	56.14	1857	60.65	299 772	77.86
Single‐parent	244	39.35	696	37.46	1022	33.38	79 388	20.62
*Sex*								
Male	448	72.26	1412	76.00	2450	80.01	196 718	51.09
Female	172	27.74	446	24.00	612	19.99	188 302	48.91
*Number of years with at least one suspected drug use offense* ^a^								
0	—	—	—	—	—	—	378 393	98.28
1	412	66.45	1089	58.61	1796	58.65	4950	1.29
2	113	18.23	414	22.28	711	23.22	1165	0.30
3	63	10.16	196	10.55	355	11.59	346	0.09
4	28	4.52	118	6.35	150	4.90	135	0.04
5	4	0.65	41	2.21	50	1.63	31	0.01
Total (*n*)	620		1858		3062		385 020	

^a^ During a 5‐year follow‐up from the year of the first drug use offense, or 2007–2011 for the general population.

Rates per 1000 person‐years of premature mortality and morbidity were significantly higher among individuals with drug‐related police contact compared with the general population (Table 2). Rates were clearly lower among the early onset group compared with the later onset groups, but the within each age‐group rate ratios in comparison to the general population were similar. The mortality rate ratio of those with a drug‐related police contact to general population was around 14 among 15‐ to 16‐year‐olds and 17‐ to 18‐year‐olds and only slightly lower (11.20) among 19‐ to 20‐year‐olds. Morbidity rate ratios were similar across age groups (12.69 among 19–20 years, 11.54 among 17–18 years and 10.84 among 15–16 years).

**Table 2:** Rates of mortality and morbidity (per 1000) for causes related to substance use or violence by age at first offense for drug use during a 5‐year follow‐up from the year of the first drug use offense, and for 2007–2011 for the general population

	Population with drug‐related police contact				General population				
Age^a^	Person‐years	Events	Rate (per 1000 person‐years)	95% CI	Person‐years	Events	Rate (per 1000 person‐years)	95% CI	Rate ratio^b^
*Mortality*				
15–16	3079	9	2.92	1.56–6.18	656 076	133	0.20	0.17–0.24	14.42
17–18	9126	48	5.26	4.00–7.07	643 188	246	0.38	0.34–0.43	13.75
20–21	15 039	76	5.05	4.06–6.38	613 878	277	0.45	0.40–0.51	11.20
Total	27 244		4.88	4.13–5.81	1 913 142	656	0.34	0.32–0.37	14.24
*Morbidity*				
15–16	2631	161	61.20	52.43–71.76	648 051	3659	5.65	5.47–5.83	10.84
17–18	7377	593	80.39	74.03–87.38	633 331	4061	6.41	6.22–6.61	12.54
20–21	11 804	1033	87.51	82.11–93.33	602 822	4156	6.89	6.69–7.11	12.69
Total	21 811	1787	81.93	78.09–85.99	1 884 203	11876	6.30	6.19–6.42	13.00

CI, confidence interval.

^a^ Age measured at first police contact in the population with drug‐related police contact and in 2007 in the general population.

^b^ Ratio of mortality or morbidity rate in population A to rate in population B.

**Figure 1:**
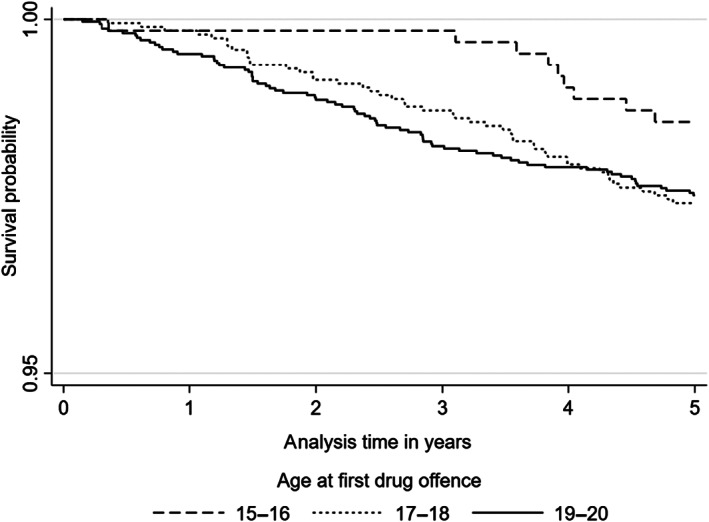
Kaplan–Meier survival plots of 5‐year mortality by onset age group.

**Figure 2:**
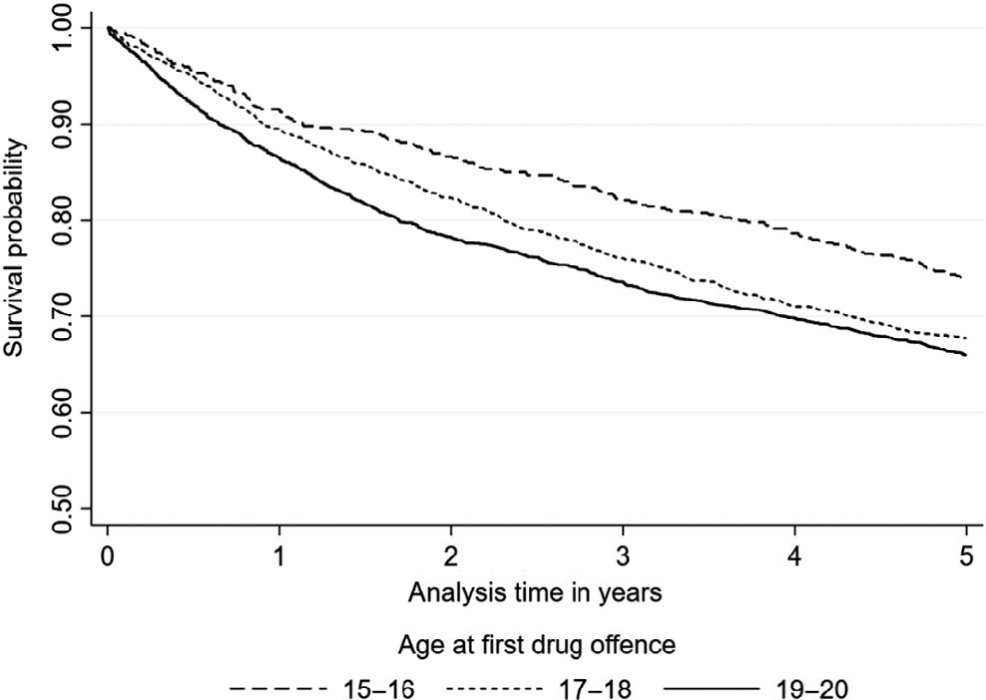
Kaplan–Meier survival plots of 5‐year morbidity by onset age group.

Based on Kaplan–Meier survival curves, the probability of survival during the follow‐up period was slightly higher among the youngest groups compared with the two other groups in both mortality (Figure 1) and morbidity (Figure 2). Deaths in particular among 15–16‐year‐olds only started to increase after 3 years since first police contact. In both mortality and morbidity, survival declined faster in the oldest age group (19–20 years), but slowed down during the 5‐year follow‐up. For morbidity, differences between age groups in the probability of survival can be seen early on in the follow‐up period, whereas for mortality differences between age groups are less clear at the first year of follow‐up. Roughly a third of the individuals with drug use‐related police contact had at least one care episode during the 5‐year follow‐up (Figure 2).

Crude hazard ratios from the Cox proportional hazards models (models 0) showed that neither premature mortality nor morbidity at ages 21–25 were associated with early onset of drug use‐related police contact. No major changes were observed when adjusting for birth year, sex and other background factors (models 1 and 2). Compared with adolescents with drug use‐related police contacts, mortality (hazard ratio 0.16, confidence interval 0.09–0.31) and morbidity (hazard ratio 0.12, confidence interval 0.11–0.15) was significantly lower among those with no police contacts due to drug use in adolescence (Table 3).

**Table 3:** Hazard ratios and 95% confidence intervals of mortality and morbidity at ages 21–25 by age at first offense for drug use^a^

				Mean time	Model 0		Model 1		Model 2	
Age at the first offense	Person years	Events, *n*	Censored, *n*	at risk, years	HR	95% CI	HR	95% CI	HR	95% CI
*Mortality*										
Drug use 15–16 (ref.)	2965	10	8	4.92	1		1		1	
Drug use 17–18	8897	44	12	4.92	1.47	0.74–2.92	1.51	0.76–3.01	1.55	0.77–3.09
Drug use 19–20	14 867	72	36	4.91	1.44	0.74–2.78	1.43	0.73–2.78	1.52	0.78–2.98
No offense below age 21	1 862 189	658	5797	4.96	0.1	0.06–0.20	0.13	0.07–0.24	0.16	0.09–0.31
Total	1 888 918	784	5853	4.96						
*Morbidity*										
Drug use 15–16 (ref.)	2435	181	13	4.04	1		1		1	
Drug use 17–18	7131	558	36	3.95	1.05	0.89–1.25	1.04	0.87–1.23	1.08	0.90–1.28
Drug use 19–20	11 851	972	61	3.92	1.1	0.94–1.29	1.07	0.91–1.26	1.16	0.99–1.40
No offense below age 21	1 829 536	12 143	6181	4.87	0.09	0.08–0.11	0.1	0.08–0.11	0.12	0.11–0.15
Total	1 850 952	13 854	6291	4.86						

^a^ Model 0: crude, Model 1: adjusted for sex, birth year and hospital district, Model 2: Model 1 + parental education, family structure at age of 14.

CI, confidence interval; HR, hazard ratio.

As mentioned in the Methods section, the Cox proportional hazards models here are subject to a modest survivor bias, since we only included individuals alive and residing in Finland at age 21. From the sample of individuals with a drug use related police contact used in calculations of rates above, 75 (1.35%) individuals died (66 due to substance use or violence) and 24 (0.43%) emigrated before the age of 21. From the general population sample, 997 (0.26%) individuals exited because of death (of which 532 due to substance use or violence) (Note: Of those 75 individuals with police contact who died before the follow‐up, 17% were age 15–16, 47% were age 17–18 and 36% were age 19–20.) and 4364 (1.1%) because of emigration before age 21. The survivor bias should be rather limited, given the low number of individuals exiting before age 21.

## Discussion and Conclusions

In this paper we analysed whether early onset of drug use‐related police contacts predicts premature morbidity or mortality. Results show high rates of mortality and morbidity due to substance use and violence among individuals with drug use‐related police contact regardless of age at first contact. The majority of adolescents with drug use‐related police contacts were males, and came from less advantaged family backgrounds compared with the general population. Most of them had multiple contacts with the police due to suspected drug use.

When comparing adolescents according to age at first police contact (15–16, 17–18 and 19–20), the mortality rate as well as morbidity rate were higher in groups with later drug use‐related police contacts compared with the youngest age group, and the probability of survival during the 5‐year follow‐up period was slightly higher among the youngest group in both mortality and morbidity. Compared with older age groups, the mortality rate among the youngest age group was especially low in the beginning of the 5‐year following period, but increased during the follow‐up period. These findings suggest that adolescents with early police contact could have a lower risk of premature mortality and morbidity. However, there was no statistically significant association between the age of drug use‐related police contacts and the hazard of mortality or morbidity at ages 21–25. When compared to general population, adolescents with drug use‐related police contact had about 10–14 times higher rates of mortality and morbidity due to substance use and violence but within each age‐group the rate ratios in comparison to the general population were similar.

Differences in mortality rates between user groups are at least partly explained by age in general and not by age of the first police contact, as differences in mortality and morbidity rates between age groups 15–16, 17–18 and 19–20 are evident also in the general population. When these age differences are taken into account, mortality rate was slightly higher among those with early onset compared with those with later onset. For morbidity, adolescents with later onset had higher rates of hospitalisation due to substance use or violence when comparing either different user groups or police contacted users to the general population. In all comparisons regardless of the age at onset, mortality and morbidity are over 10 times higher among adolescents with a police contact for drug use compared with the general population showing that overall risk of severe health consequences in all groups of drug use onset is high.

The findings challenge earlier results [6] suggesting that early drug use onset increases the risk of premature mortality, which could suggest that longitudinal population‐level register data provides additional insight into the phenomena compared with samples derived from clinical programs. Earlier studies used the age of 18 as the cutoff for early vs. late onset, when our study includes more detailed age categories of onset. This could mean that drug use may be less problematic in early adolescence compared with later adolescence and that 15–16 years olds may still be influenced more by parents social characteristics. Alternatively, earlier drug use may be more random as users may stop or substitute legal substances. The finding, that a smaller proportion of adolescents in the younger age group had multiple police contacts due to drug use compared with adolescents in the older age groups, supports this view. Along with parents, child protective services can influence younger adolescents more than older ones. Overall, there seems to be no evidence of a linear influence (younger onset—higher risk) as suggested in earlier work [6].

However, it should be taken into account that the measure used here—drug‐related police contacts—is limited in measuring drug use onset. The first time one gets caught by the police for using drugs is likely not the first time they actually use drugs (or could be diagnosed with a substance use disorder). It is argued that in Nordic countries police records separate quite well heavy drug users from more occasional or recreational users, while the majority of those with high consumption of illegal drugs have a high probability of getting caught or committing other crimes [4]. This study focuses on all types of unlawful drug use, including the occasional party use, which likely increases the gap between registered use and actual use for occasional users compared with heavy drug users suggesting that register data derived from authority contacts may emphasise the effects of getting caught rather than the actual effects of drug using. This should be studied further to better understand how well register data can capture actual use rates. Even with this limitation, population‐level analysis brings a novel approach to the discussion without the generalizability problems found in alternative research strategies.

In addition, societal context has an influence on the wellbeing of drug users. In Finland, underaged police contact means automatic intervention by social services in addition to the criminal process (or only intervention, if the child is under 15 [16]). For offenders over 18, this does not occur. Therefore, it is possible that some of the findings in this study might be explained by an earlier contact with social services. Although assessing interventions and the role of health and social services in the promotion of drug users wellbeing is beyond the scope of this study, the study suggests that early police contact at least in the Finnish context could be beneficial.

Some limitations need to be taken into account when interpreting the findings. First, detailed information on the characteristics of drug use, including the type, start dates, if the use was one‐time or ongoing, and how serious or heavy (dosage and frequency) of the use was not available in the register data. In addition, administrative register data include only individuals with a treatment or police contact, excluding some drug users, although the latter proportion has been evaluated to be rather small in Nordic welfare states [4, 10]. Despite these limitations, administrative data have major strength for this type of study, including large sample size and a long follow‐up, and no recall or non‐response biases. Therefore, it is safe to conclude that many adolescents with drug use‐related police contacts come from potentially challenging societal background and they have high risk of mortality and morbidity regardless of the age of drug onset.

## Conflict of Interest

The authors have no conflicts of interest.

## Appendix A

Coding of deaths and inpatient and specialised outpatient care due to substance use or violence followed the 10th revision of the International Classification of Diseases (ICD). Substance use‐related admissions were identified using both main and subsidiary diagnoses.

- Mental and behavioural disorders due to psychoactive substance use

ICD‐10: F10–F16, F18–F19

- Poisoning by drugs, medicaments and alcohol

ICD‐10: T36–T51, X44–45, X40–X45

- Intentional self‐harm

ICD‐10: X60–X84, Y870, Y87

- Assaults

ICD‐10: X85–Y09, Y871
